# Evaluation of hop tests based on Y-Balance test and FMS test outcomes in volleyball and basketball players to identify those prone to injury: a potential predictor of injury

**DOI:** 10.1186/s13102-024-00976-5

**Published:** 2024-09-07

**Authors:** Sepideh Latifi, Zeinab Kafshgar, Atiye Yousefi

**Affiliations:** 1 Department of Sports Injury and Biomechanics, Faculty of Sport Sciences and Health, University of Tehran, Tehran, Iran; 2 Faculty of Sport Sciences and Health, University of Tehran, Tehran, Iran

**Keywords:** Functional movement screen (FMS), Hop tests, Y-Balance test (YBT), Sports injuries prediction, Volleyball, Basketball

## Abstract

**Background:**

The quest for a reliable and effective method to identify athletes at risk of injury holds the promise of significantly reducing injury rates and improving overall athletic performance. This research delved into the relationship between the Functional Movement Screen (FMS), Y-Balance Test (YBT), and Hop tests (Side hop, Medial triple hop, and Lateral step-down hop), aiming to determine the potential in predicting injuries of hop tests among division 1 volleyball and basketball players.

**Methods:**

This research was conducted with fifty-two participants from the Division 1 league, encompassing both volleyball and basketball players. The study rigorously employed the Functional Movement Screen (FMS), the Y-Balance Test (YBT), and various hop tests (side hop test, medial triple hop test, lateral step-down hop) to measure relevant variables. The data analysis used logistic regression, ensuring a comprehensive approach to the study.

**Results:**

Results showed no significant relationship between FMS and Hop test for predicting injuries, but there was a relationship between ΔY and side hop that shows side hop test can predict injury, but there was no relationship between Lateral step down, Medial triple hop, and ΔY.

**Conclusions:**

Based on our findings, side hop, despite the medial triple hop and lateral step-down test, can be used as a sports injury predictor.

**Supplementary Information:**

The online version contains supplementary material available at 10.1186/s13102-024-00976-5.

## Background

Volleyball and basketball, two of the most popular sports globally, offer many benefits to athletes. These include improved body composition, cardiorespiratory function, increased strength, enhanced self-esteem, and positive psychosocial well-being. However, it’s essential to be aware that these sports also place significant physical demands on athletes. During games and training, players engage in repetitive jumps, sudden changes in direction, running, and rapid deceleration. As a result, both volleyball and basketball players face a heightened risk of musculoskeletal injuries. Athletes need to know they are not alone in their experiences, as injuries such as ankle sprains, knee injuries, and shoulder injuries are common in both sports [1, 2]. Musculoskeletal injuries are among those death incompetencies that tend to reduce athletes’ sports life. This has led researchers and sports medicine specialists to investigate sufficient ways to identify risk injury factors to diminish the incidence rate of injuries [3].

The well-known saying ‘Prevention is better than cure’ has existed for a while. Sports injuries don’t usually result from a single cause but rather from a combination of intrinsic (related to the individual) and extrinsic (environmental) factors. The van Mechelen model emphasizes early intervention to reduce risk factors before injuries occur. It’s worth noting that researchers at the forefront of sports injury prevention recognize the importance of understanding these risk factors and injury mechanisms [4]. Some inherent factors, such as a history of previous injuries, loose ligaments, limited range of motion, inadequate aerobic fitness or muscle strength, poor balance, and infrequent physical activity, can increase the likelihood of sports-related injuries [5].

Evaluating functional performance is a method for identifying athletes susceptible to sports-related injuries [6]. Considering the fact that targeting high-risk athletes through screening programs proves more beneficial than a universal intervention approach [4], Establishing an effective and suitable technique to identify athletes at a heightened risk of injury can result in developing intervention strategies. These strategies are designed to lower the occurrence of injuries and enhance the overall performance of the athletes [1]. FMS and YBT tests are examples of field-based measurements used productively by sports medicine professionals to evaluate functional performance [7].

Sports medicine professionals advocate for a set of clinical field-based screening tools, including the Y Balance Test (YBT), Star Excursion Balance Test, Landing Error Scoring System (LESS), Functional Movement Screen (FMS), and hopping tests. These tools serve as user-friendly alternatives to laboratory-based measurements [2, 3]. These clinical screening tests can identify risk factors associated with musculoskeletal injuries, such as neuromuscular control/imbalance and poor core stability and strength [5, 6]. Considering these factors, the tests serve as diagnostic instruments, particularly within team sports contexts. As a result, the outcomes of the injury prevention program tests can be applied following the findings from athlete assessments [4, 5, 7].

As a screening tool, FMS can identify compensatory movement patterns, which influence optimal functions [5]. FMS is an efficient and credible method to distinguish motion deficits and body asymmetry [8, 9, 10]. FMS has demonstrated strong inter-rater and intra-rater reliability. It effectively identifies impaired movement patterns and motor skills, which are believed to be indicators of injury risk, providing reassurance about its benefits [9, 11–13].

On the other hand, deficiencies in dynamic neuromuscular control of the lower extremities have been recognized as a risk factor for injuries [14, 15]. YBT is a reliable tool for assessing dynamic balance and neuromuscular control. During the test, individuals stand on one leg, requiring strength, flexibility, and proprioception. YBT performance benefits sports training and reflects an athlete’s readiness for participation in sports. Any flexibility, strength, or power limitations can affect fundamental sports movements [8].

Moreover, Hop testing is a powerful predictor, frequently proposed as a practical, performance-based measure. It reflects neuromuscular control, strength, and limb confidence. Importantly, it requires minimal equipment and administration time. Researchers have suggested that hop tests can predict future knee-related problems and serve as evaluative tools for treatment response. These tests simulate dynamic knee stability during sports activities, preparing patients for a safe return to play [16]. They are a foundation for return-to-sport assessments to achieve acceptable standards (such as a Limb Symmetry Index of ≥ 90%) during rehabilitation. Some research indicates that lower-limb functional test scores assessed during and after ACL rehabilitation correlate with future knee-related outcomes. For instance, poor hop test performance is linked to worse quality of life in the future, and fewer successful one-leg rises are associated with the development of radiographic osteoarthritis. However, other studies have not found a consistent association between functional performance and future outcomes [17].

Existing research has primarily focused on hop tests as predictors of re-injury. However, little attention has been given to their potential as sports injury predictors. We see this as gaps in screening tests and predicting injury fields. Researchers proved that hop tests are practical as sports re-injury predictors, and they assess dynamic balance and stability in lower limbs [16]; we hypothesized they could also be practical tests for predicting and preventing injuries. This tool offers physical therapists, athletic trainers, coaches, and other clinicians valuable information. It equips them with the necessary insights to mitigate injury risks in volleyball and basketball players. Identifying at-risk athletes in pre-season screening using a low-cost, time-efficient, and low-physical-risk screen can potentially decrease injuries and medical costs to athletes, offering a hopeful prospect for cost savings in Athlete Your practice.

Our study aims to investigate whether hop tests (side hop test, medial triple hop, lateral step-down hop) can reliably predict injuries in volleyball and basketball players. Given the high incidence of lower extremity injuries in these sports, understanding the predictive value of these tests is crucial. We hypothesize that individuals with lower Functional Movement Screen (FMS) scores (< 14), ΔY ≥ 4 will also exhibit lower hop test scores. Ultimately, this study seeks to determine the predictive value of hop tests based on FMS and YBT outcomes. The potential impact of this study on injury prevention in sports is significant, and we hope it will inspire further research and initiatives in this field, providing you with valuable insights to enhance your practice.

## Methods

The present correlational descriptive research plans to randomize data sampling, which is predictive and practical.

### Participant

Fifty-two participants (35 females, 17 males, mean age 20.94 ± 2.37) from volleyball (*n* = 40) and basketball (*n* = 12) division 1 league were involved.

### Procedures

Before data collection, eligible participants were invited to the Biomechanics lab at the University of Tehran’s Sport Sciences Department. Participants self-reported their height and weight, and their BMI was calculated. We assessed lower-limb dominance based on their preferred kicking leg. Bilateral leg length measurements in centimeters were used to normalize reach distances, as leg length has been shown to impact YBT performance [18]. Each session was conducted privately to respect participants’ privacy. Individual testing sessions lasted 30 min. All assessments were measured in the evening (between 4:00 p.m. and 6:00 p.m.). Participants were randomly assigned to start testing with the screening tests (FMS, YBT, Hop test). All participants wore sportswear. For YBT measurements, they were barefoot, while for the FMS and Hop tests, they wore shoes. Participants received oral briefings about the study’s objectives, risks, and benefits before data collection. They were informed of their rights as human participants, and informed consent was obtained following ethical approval from the University of Tehran’s Ethics Committee research (Ethics Approval Code: IR.UT.SPORT.REC.1402.078) which was crucial in ensuring the research’s adherence to ethical standards. Throughout the study, we prioritized contributors’ rights and safety, securing written permissions for participant images.

### Data collection tools

The study involved five assessments for each participant, including the FMS, YBT, and various Hop tests (Side hop, Medial triple hop, and Lateral step-down hop). Our research on the potential predictor of Hop tests based on the FMS score and ΔY is significant in injury prevention. By categorizing participants into two groups based on the FMS cut-off point score (score < 14 susceptible to injury, score ≥ 14 not susceptible to injury) [19] and the ΔY cut-off point score (score ≥ 4 susceptible to injury, score < 4 not susceptible to injury), we can confidently predict injury susceptibility. This reassures us of the reliability and applicability of our research findings in sports medicine and injury prevention [20].

FMS includes seven distinct experiments that evaluate fundamental movement patterns, encompassing the deep squat, hurdle step, in-line lunge, shoulder mobility assessment, active straight-leg raise, trunk stability push-up, and rotary stability tests [21].

The FMS is a comprehensive tool for understanding the body’s movement function. It assesses seven fundamental movement patterns related to balance, mobility, and stability, which are essential for proprioception and kinesthetic awareness. Each FMS component directly challenges the body’s cohesive kinetic function. Ratings range from zero to three: zero indicates pain during the movement, one signifies poor performance or inability to complete the task, two denotes compensatory movement patterns, and three represents excellent performance. The composite score, obtained by summing up ratings for all seven components, is a powerful tool for identifying injury risk. A composite score below 14 is a clear sign of a high risk of injury [7, 9, 10, 22]Participants were in the preseason. They had a 5-minute general warm-up. Then, we taught them the instructions for performing tasks, and after one practice attempt, we recorded their next effort.

The YBT, an objective tool for assessing balances in functional movement [11]. Involves individuals positioning themselves at the center of the YBT kit. They are then asked to extend their reach as far as possible, ensuring their feet remain on the reach indicators. After each stretch, they revert to the starting point. This process is repeated in anterior, posterolateral, and posteromedial directions. The test is conducted on both legs [12, 21]. The furthest point of the foot, typically the toe, touching the measurement stick signifies the maximum reach distance. When balance is lost, feet are placed on top of the measurement indicator, or the indicator is kicked for additional points, which are classified as failed attempts. The composite score is derived from the measured distances in the anterior, posteromedial, and posterolateral directions, ensuring the objectivity of the results. Subsequently, a percentage is calculated by dividing the total by thrice the length of the participant’s limb and multiplying the result by 100 [13, 21]. The length of the limb is determined using a tape measure, with the individual in a supine position from the anterior superior iliac spine to the most distal aspect of the medial malleolus. The individual’s preferred leg for kicking a ball is accounted for to identify the dominant lower extremity [12].

Participants were in the preseason. All of them had a 5-minute general warm-up. Then, we taught them the instructions for performing tasks, and after one practice attempt, we recorded their three subsequent successive attempts. It has been proposed that a reach difference of equal to or more than 4 cm between the dominant and non-dominant legs could increase the risk of injury [14, 20].

The hop tests demonstrated variations in performance between injured and non-injured limbs [4, 15]. In establishing a score for secure Return to Play (RTP), the existing literature relies on a Limb Symmetry Index (LSI) benchmark of 90% [4, 16]. A decrease in LSI asymmetry might be linked to an enhancement in RTP rates and a reduction in re-injury occurrences [4, 23]. Hop tests also demonstrated high reliability, as evidenced by intra-class correlation coefficients (ICCs) ranging from 0.84 to 0.97 [4, 15]. During the medial triple hop test, the participant is instructed to position themselves on the leg being tested, with the medial aspect of the foot aligned perpendicularly to the direction of the hop [24]. The participant performed three successive hops on the same leg, aiming to land as distantly as possible in the direction of a rolling meter (aligned with the medial direction of the standing leg). The foot’s orientation was required to stay perpendicular to the direction of the hop. The cumulative distance of the three consecutive hops was gauged from the medial aspect of the foot at the point of take-off to the medial aspect of the foot upon landing [19]. In the side hop test, participants were coached to execute one-legged hops, moving laterally beyond two parallel tape strips 40 cm apart. The goal was to accomplish the maximum number of hops within 30 s. This test was challenging due to its multifaceted nature, requiring endurance, muscular strength, and stability [25, 26]. Finally, in the lateral step-down hop test, participants were positioned on a step that was 15 cm high. They were then guided to touch the ground and return to the initial position, repeating this action for 30 s on each leg.

Participants were in the preseason. All of them had a general warm-up of 5 min.

In every test, it was essential for participants to maintain balance upon landing before the hop distance could be measured and documented. Any instance of the second foot touching down during the hop or landing or any irregular movement causing the intended foot to shift upon landing was deemed a failed attempt. If the hop was not executed correctly, attempts were repeated until a successful hop was achieved. Each limb underwent two hops rounds, with a brief rest period. Participants were allowed one practice attempt before each hop test [4].

### Statistical analysis

A binary logistic regression (α = 0.05) test was used to predict participants’ susceptibility to injury to identify the potential predictor of hop tests (Side hop, Medial triple hop, Lateral step-down hop) based on FMS and YBT outcomes. Hop tests were used to assess the correlation for anticipated injuries based on FMS and YBT scores, Regression analysis is a statistical technique used to measure the relationship between variables predicting future values [27].

All binary logistic regression test assumptions were met to check out the condition of binary logistic regression, including the presence of a dichotomous outcome variable, the absence of multicollinearity, and the lack of enormously influential outliers.

The susceptibility to injury was dichotomized as yes or no. Descriptive statistics for demographics and clinical variables were expressed as means with standard deviations (SD) for continuous variables and counts (n) with percentages for categorical variables. To examine the association between scores of the Hop tests (side hop, medial triple hop, lateral step-down hop) and the susceptibility to injury (based on FMS and YBT outcomes), binary logistic regression was used with odds ratios (OR) and 95% confidence intervals (95% CI). SPSS 21 statistical software performed all statistical calculations. An alpha level of 0.05 was used for analysis.

## Results

The description of the variables, i.e., height, weight, body mass index, YBT, and Hop of the participants, are presented in Table 1. The findings showed that 35 (67.3%) of the participants were female,17 (32.7%) were male, and the dominant foot of 48 (92.3%) of the participants was the right foot, while the dominant foot of 4 (7.7%) of the participants was the left foot. According to the FMS cut-off point score, 11 (21.2%) were susceptible to an injury, while 41 (78.8%) were not.

**Table 1:** Demographic characteristics and variables

Demographic Characteristics and Variables	Mean	± SD
Height	170.8	± 10.8
Weight	62.5	± 10.97
BMI	21.56	± 2.35
FMS	15.76	± 2.37
Y right	95.40	± 15.52
Y left	94.62	± 16.56
∆Y [=Y_Left_-Y_right_]	5.62	± 5.42
Y Dominant foot	94.68	± 14.95
Side Hop right	29.05	± 15.06
Side Hop left	28.03	± 16.13
Medial triple Hop right	305.03	± 64.18
Medial Triple Hop left	303.05	± 72.05
Lateral Step Down right	19.51	± 7.45
Lateral Step down left	19.63	± 8.35

To identify the potential predictor of hop tests (Side hop, Medial triple hop, Lateral step-down hop) based on FMS scores, binary logistic regression results indicated that hop tests (Side hop, Medial triple hop, Lateral step-down hop) were not significant predictors of FMS scores (*P* > 0.05) (See Table 2).

**Table 2:** Logistic regression results. Prediction of hop tests (side hop, medial triple hop, and lateral step-down hop) based on FMS

	B	S.E.	Wald	df	Sig.	Exp(B)	95.0% C.I.for EXP(B)	
							Lower	Upper
Side hop R	0.027	0.025	1.140	1	0.286	1.027	0.978	1.080
Constant	0.575	0.737	0.609	1	0.435	1.777		
Side hop L	0.075	0.035	1.205	1	0.033	1.077	1.006	1.154
Constant	− 0.415	0.772	0.289	1	0.591	0.661		
Medial Triple hop R	0.005	0.006	0.680	1	0.410	1.005	0.994	1.015
Constant	− 0.045	1.658	0.001	1	0.978	0.956		
Medial Triple hop L	0.006	0.005	1.256	1	0.262	1.006	0.996	1.016
Constant	− 0.413	1.540	0.072	1	0.788	0.661		
Lateral step-down R	0.13	0.082	2.836	1	0.092	1.148	0.978	1.347
Constant	-1.13	1.404	0.656	1	0.418	0.321		
Lateral step-down L	0.055	0.057	0.927	1	0.336	1.056	0.945	1.181
Constant	0.292	1.071	0.074	1	0.785	1.339		
Medial Triple Dominant	− 0.003	0.005	0.302	1	0.582	0.997	0.987	1.008
Constant	2.217	1.691	1.719	1	0.190	9.176		
Lateral Step-down Dominant	− 0.009	0.023	0.167	1	0.683	0.991	0.948	1.036
Constant	1.589	0.763	4.343	1	0.037	4.901		
Side Hop dominant	− 0.010	0.042	0.056	1	0.812	0.990	− 0.010	0.042
Constant	1.511	0.898	2.832	1	0.092	4.532	1.511	0.898

**Table 3:** Logistic regression results. Prediction of Hop Tests (Side hop, Medial triple hop , Lateral step-down hop) based on ∆YBT

	B	S.E.	Wald	df	Sig.	Exp(B)	95.0% C.I.for EXP(B)	
							Lower	Upper
Medial triple hop	− 0.008	0.005	3.197	1	0.074	0.992	0.982	1.001
Constant	2.839	1.493	3.616	1	0.057	17.093		
Side hop	− 0.062	0.023	7.378	1	0.007	0.940	0.900	0.983
Constant	2.020	0.721	7.850	1	0.005	7.539		
Lateral step-down hop	− 0.024	0.037	0.420	1	0.517	0.977	0.909	1.049
Constant	0.696	0.770	0.819	1	0.365	2.007		

The binary logistic regression test results indicated that the side hop significantly predicted ΔYBT scores OR = 0.94 (95% CI 0.90–0.98). However, the Lateral step-down hop and Medial triple hop were not significant predictors of ΔYBT scores (see Table 3).

## Discussion

The current study aimed to determine the predictive value of hop tests (Side hop, medial triple hop, and lateral step-down) based on FMS and YBT outcomes in division 1 volleyball and basketball players. This study can significantly impact the field since, to the best of our knowledge, it is the first to investigate the predictor potential of Hop Tests in Division 1 volleyball and basketball players. We hypothesized that the Hop tests would be associated with injury risk, findings that could lead to improved injury prevention strategies and player performance.

The findings revealed no significant relationship between FMS and the Hop tests for predicting injuries, opening avenues for future research. FMS includes seven distinct experiments involving the lower and upper extremities, in contrast to the Hop tests involving the lower extremities. We assumed that this difference between FMS and hop tests could be why there was no relationship between them for predicting injuries. Khalid M. Alkhathami’s research findings are intriguing, as they reveal unexpected connections and disparities. For instance, the study found a connection between FMS scores and hop test performance in college football players, with those scoring higher able to hop a greater distance, but not necessarily faster. Equally surprising was the lack of a link between FMS scores and hip or knee strength [28]. This suggests that athletes with good FMS scores do not necessarily have stronger knees and hips; strength is a factor evaluated in hop tests [29]. However, it may be rational to assume that we didn’t find a connection between the FMS and the Hop tests because of the varying demand in terms of speed. The side hop test is a valid and reliable to assess strength resistance under a fatigue state through controlled, fast, and repetitive lateral jumps [30]. On the other hand, for FMS, speed wasn’t a factor. Participants didn’t have to hurry; they could take their time; interestingly Athletes with higher FMS scores do not necessarily have better speed proved by [31] that investigated the association between speed and FMS with two speed-based outcomes, which found moderate negative associations between repeated sprint ability best time (*r* = − 0.58, *p* < 0.001) for total FMS, as well as the weak negative association between total FMS© and 5 m sprint time (*r* = − 0.13, *p* < 0.05). This highlights the urgent need for further research in the field, as it suggests that the relationship between FMS and the Hop tests is more complex than previously thought.

In this study, we conducted a series of tests, including the Side hop test, Lateral step-down, Medial triple hop, and the YBT, to investigate their relationship. A significant finding of this study is the unique and intriguing relationship between the Side hop test and a measurement we refer to as ‘ΔY.’ This relationship suggests the predictor potential of the side hop test. We assumed the reason could be that YBT and side hop tests assess dynamic balance and stability in lower limbs [16, 28]. Moreover, YBT is an evaluation that evaluates performance in three directions (anterior, posteromedial, and posterior). The total score is the combination of three directions [27] and Side hop that typically involves jumping in two directions: medial (towards the body’s midline) and lateral (away from the midline); participants had to maintain balance and control movement in both directions [25, 26]. Consequently, on both tests, medial and lateral aspects of the body are involved despite the lateral step down and medial triple hop not involving multidirectional movement. We assumed that side hop is related to ΔY despite FMS because YBT evaluates lower limb strength and control [28], but FMS evaluates lower limb and upper limb movements.

Our study’s findings underscore the need for further research. We urge researchers to investigate the relationship between each direction of the YBT separately and the lateral step-down and medial triple hop. This could lead to a deeper understanding and more accurate predictions from these tests, potentially opening new avenues for improving physical performance and injury prevention. The potential for these findings to lead to improved strategies in these areas is not just promising but also should give hope for future advancements, instilling a sense of optimism and hope in our readers.

## Conclusion

FMS, YBT, and hop tests are assessment instruments employed by practitioners in sports medicine to gauge strength, equilibrium, and movement behaviors. Although these tests evaluate fundamental concepts like dynamic stability and movement synchronization, the results of this study did not show any significant correlations between FMS and hop tests in either gender. this could be due to the use of speed in hop tests, which is not a factor in FMS. Additionally, hop tests focus on the lower limb, while FMS evaluates the upper and lower limbs. However, the result shows a significant relationship between ΔY and side hop, indicating the promising potential of the side hop test as a predictor. This potential is not just promising; it’s intriguing, as the YBT and hop test involve similar movements of the lower limbs in multiple directions, engaging both the medial and lateral aspects of the body. Moreover, we believe that since the lateral step-down hop involves lateral movement, the medial triple hop test involves medial movement, not multidirectional, the same as YBT. Moreover, despite YBT, the lateral step-down and medial triple hop do not involve multidirectional movement. The result shows no relationship between ΔY and medial triple hop and lateral step-down.

Our research demonstrates the side hop test’s predictive potential. This finding is valuable, and we strongly recommend further investigation with a larger homogenous group to establish a cut-off point for the side hop test. This ongoing research will enhance its practical application, and we invite you to be an active part of this journey. Your involvement will advance our understanding of this field, making you an integral part of the research process.

In conclusion, this study underscores the importance of employing a variety of field-based assessments by sports medicine experts and strength and conditioning trainers. These assessments are crucial for evaluating athletes’ movement patterns and physical performance abilities. Our findings, particularly the potential of the side hop test as a predictor, provide valuable insights for these professionals, potentially influencing their assessment strategies and training programs.

### Limitations

The number of participants was limited. A larger sample size would have provided more accurate data. Our tests were complex, involving a combination of physical, cognitive, and skill-based tasks. This complexity underscores the rigorous nature of our research, ensuring the highest quality of data and the need for healthy and physically fit participants to complete them. We had to take tests on one day since access to participants was difficult, and taking different and complex tests would prolong the test time. Consequently, another limitation is that the amount of resting time between the tests was limited. Also, our research was a retrospective study, and there was not any follow-up.

### Strengths

Our research, which included participants from the basketball and volleyball communities, collectively consisting of millions of players worldwide, utilized three tests, two of which are proven predictors. Moreover, our research established a relationship between these tests, an assessment that has not been done by other research in other studies. These tests were employed to assess the functional capabilities of athletes, and our study placed particular emphasis on the accuracy of these tests in predicting athletic performance, providing a solid foundation for our findings. Also, our research encompassed male and female participants, focusing on professional volleyball and basketball athletes, ensuring our findings’ reliability and validity, thereby providing a solid basis for future research and practice.

## Practical applications

Our research has shown that the side hop tests can be used as sports injury predictors. Thus, this test can be a useful screening tool to help predict injuries and make an effective prediction strategy for the athlete.

## Electronic supplementary material

Below is the link to the electronic supplementary material.

Supplementary Material 1

Supplementary Material 2

## Data Availability

Data is provided in the supplementary information files.

## Abbreviations

YBT: Y Balance Test
LESS: Landing Error Scoring System
FMS: Functional Movement Screen
SEBT: Star Excursion Balance Test
YBT-LQ: Y-Balance Test Lower Quarter
RTS: Return to Sport
LSI: Limb Symmetry index
